# Supplementary material for: Stable Isotope Analysis Reveals Common Teal (Anas crecca) Molting Sites in Western Siberia: Implications for Avian Influenza Virus Spread
Source: Microorganisms. 2024 Feb 9;12(2):357. doi: 10.3390/microorganisms12020357 (PMC10891923; doi:10.3390/microorganisms12020357)
Supplement: Supplementary file 1 [file microorganisms-12-00357-s001.zip › Figure S9.pdf]

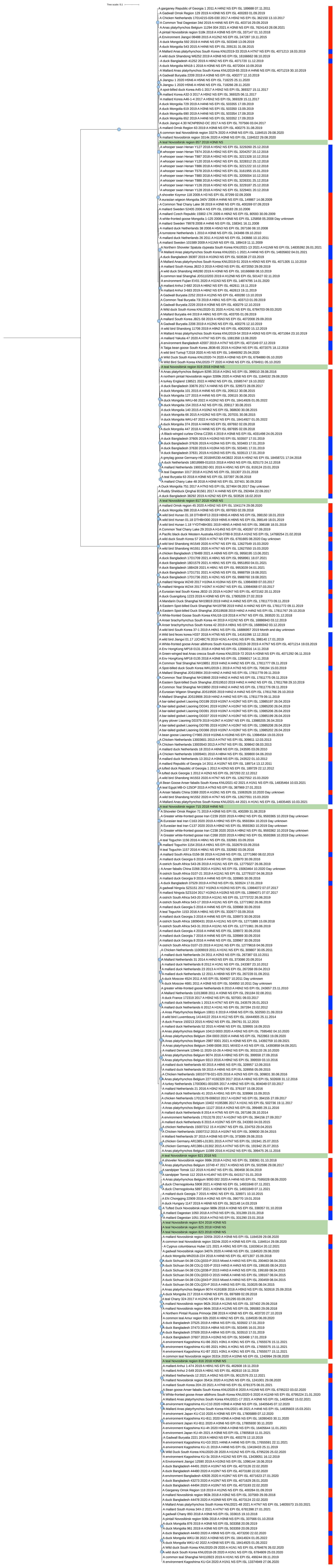
Tree scale: 0.1
