# Supplementary figures and images for: Stable Isotope Analysis Reveals Common Teal (Anas crecca) Molting Sites in Western Siberia: Implications for Avian Influenza Virus Spread
Source: Microorganisms. 2024 Feb 9;12(2):357. doi: 10.3390/microorganisms12020357 (PMC10891923; doi:10.3390/microorganisms12020357)

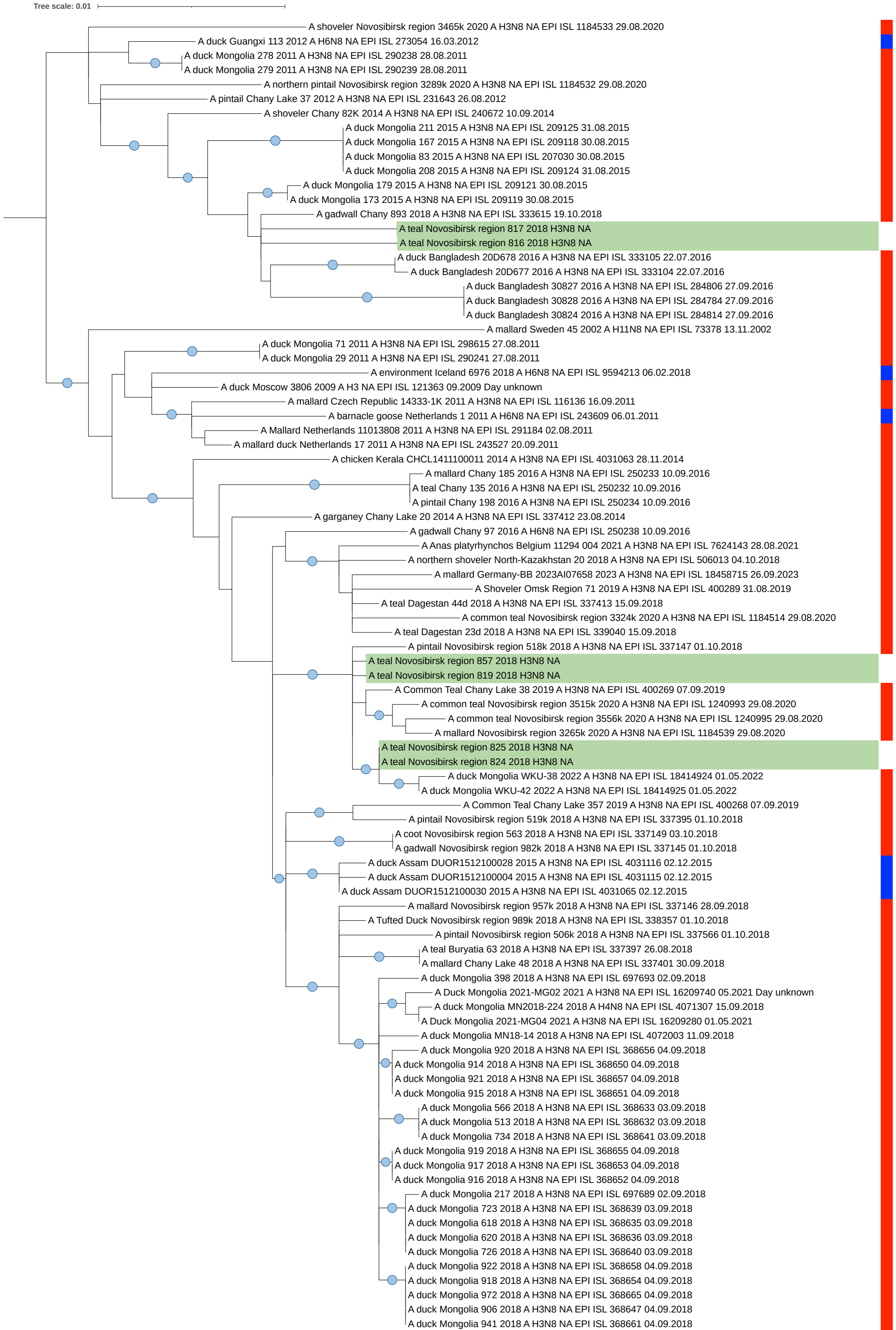

Supplement: Supplementary file 1 [file microorganisms-12-00357-s001.zip › Figure S1.pdf]

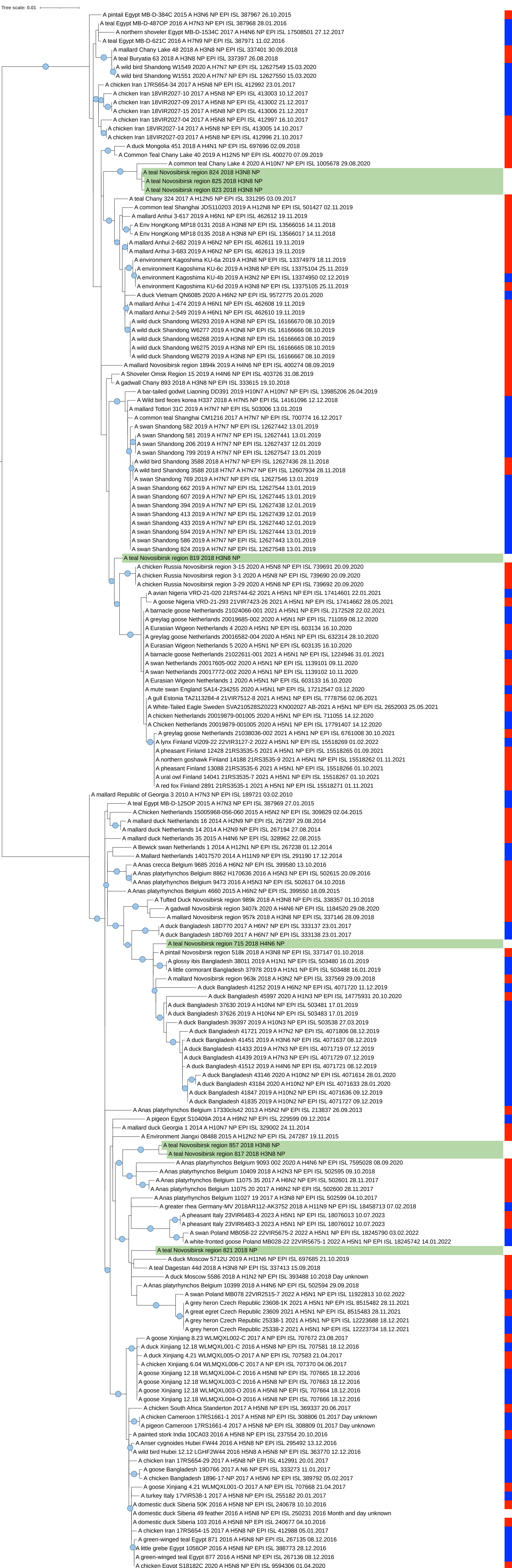

Supplement: Supplementary file 1 [file microorganisms-12-00357-s001.zip › Figure S10.pdf]

Tree scale: 0.01

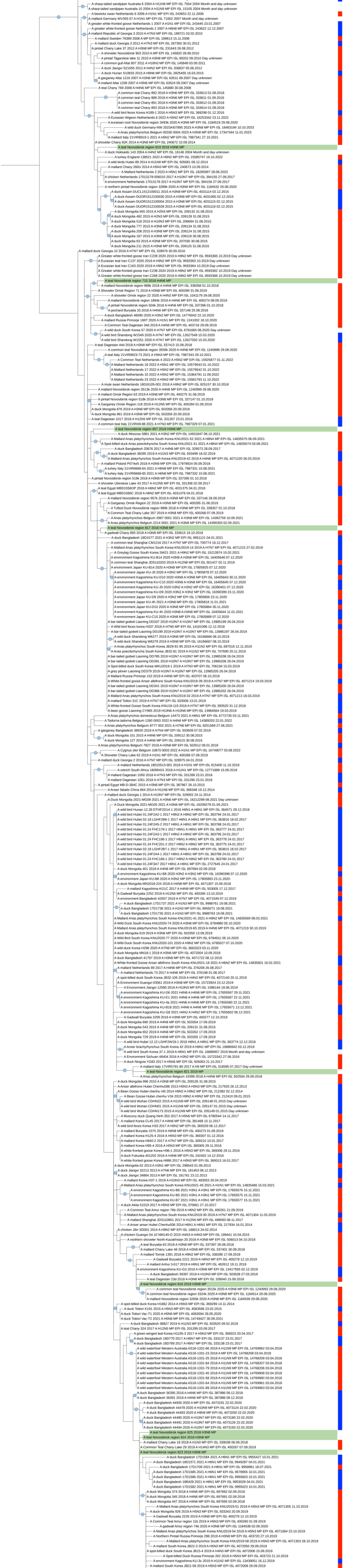

Supplement: Supplementary file 1 [file microorganisms-12-00357-s001.zip › Figure S11.pdf]

Tree scale: 0.01

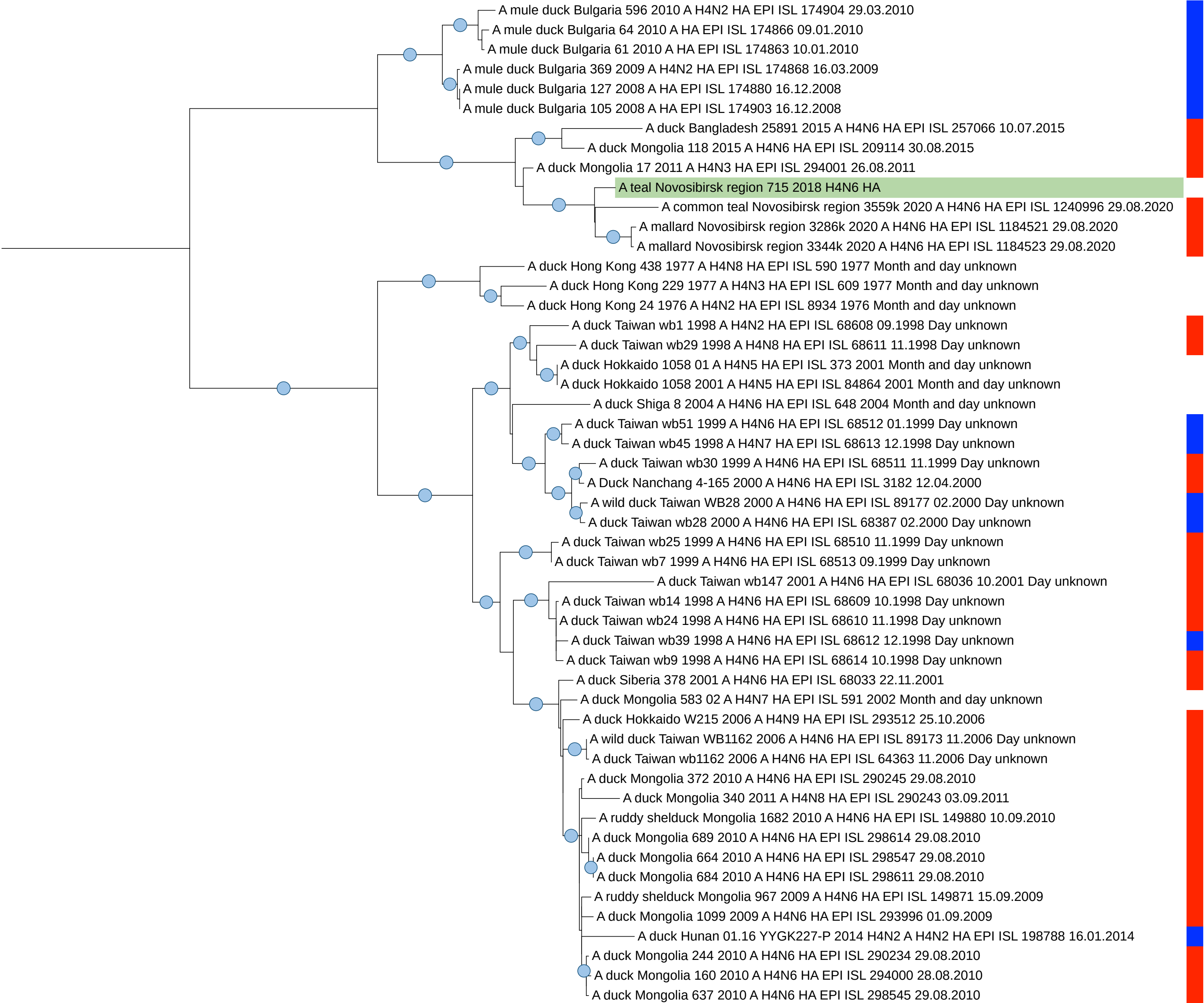

Supplement: Supplementary file 1 [file microorganisms-12-00357-s001.zip › Figure S2.pdf]

Tree scale: 0.01

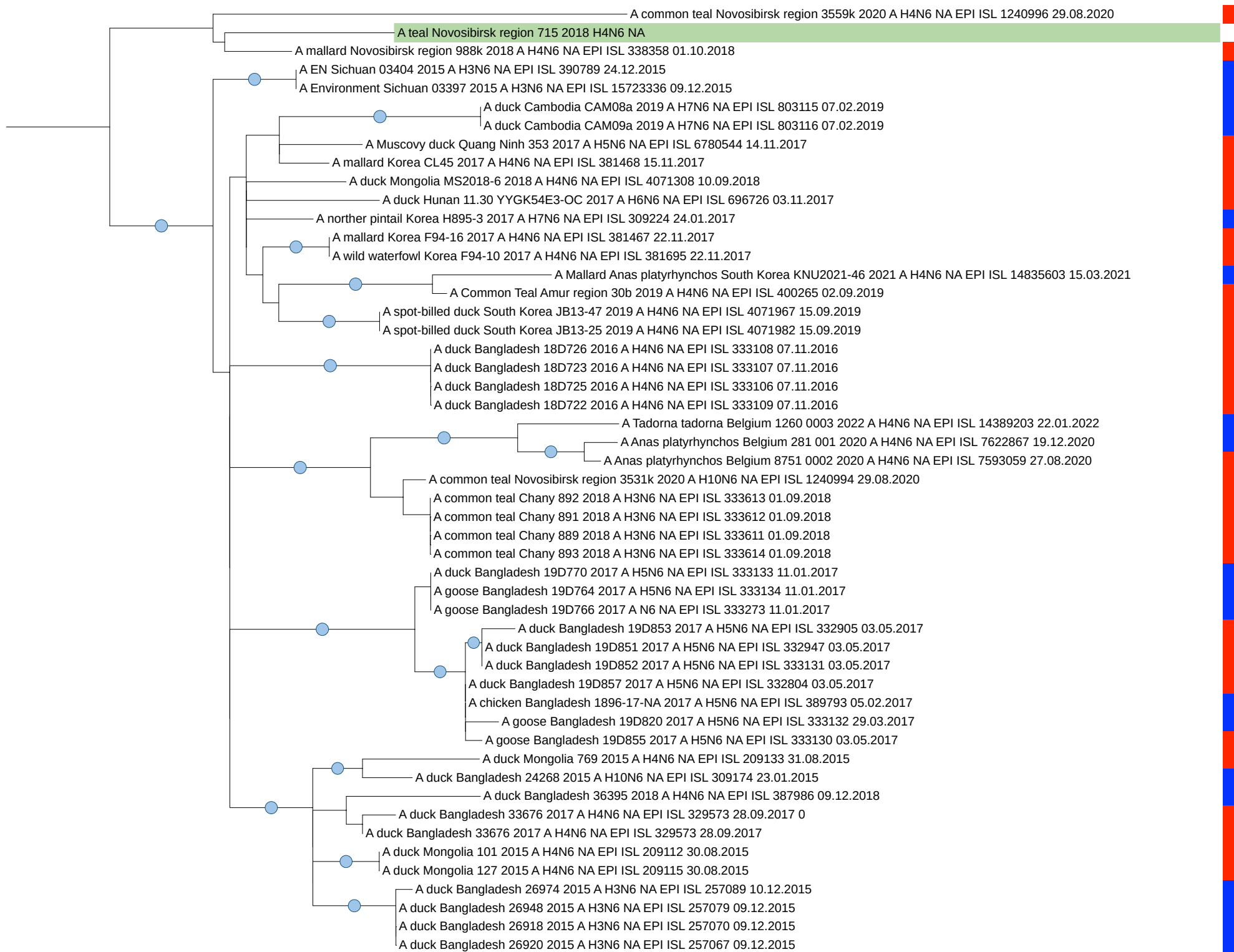

Supplement: Supplementary file 1 [file microorganisms-12-00357-s001.zip › Figure S3.pdf]

Tree scale: 0.01

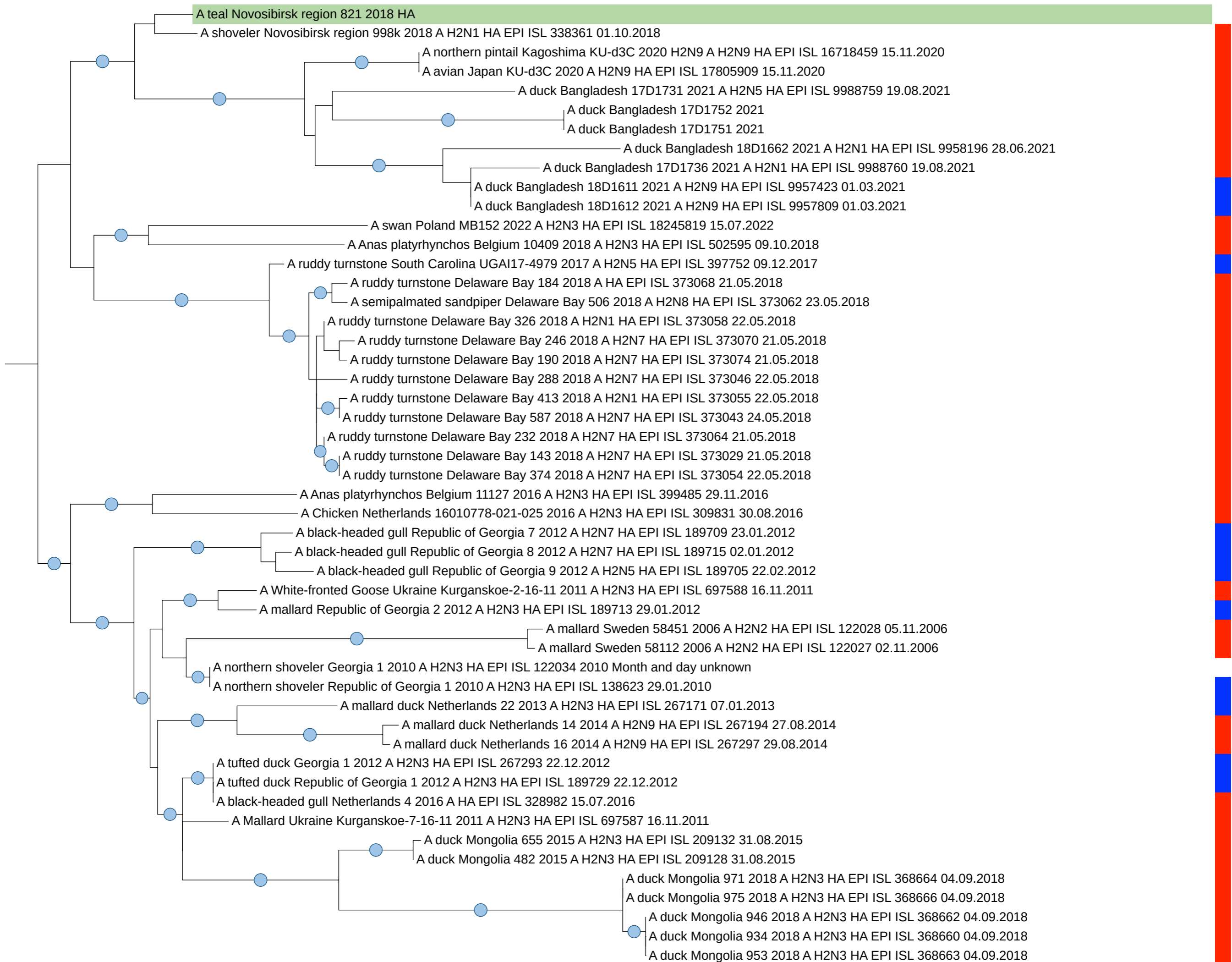

Supplement: Supplementary file 1 [file microorganisms-12-00357-s001.zip › Figure S4.pdf]

Tree scale: 0.01

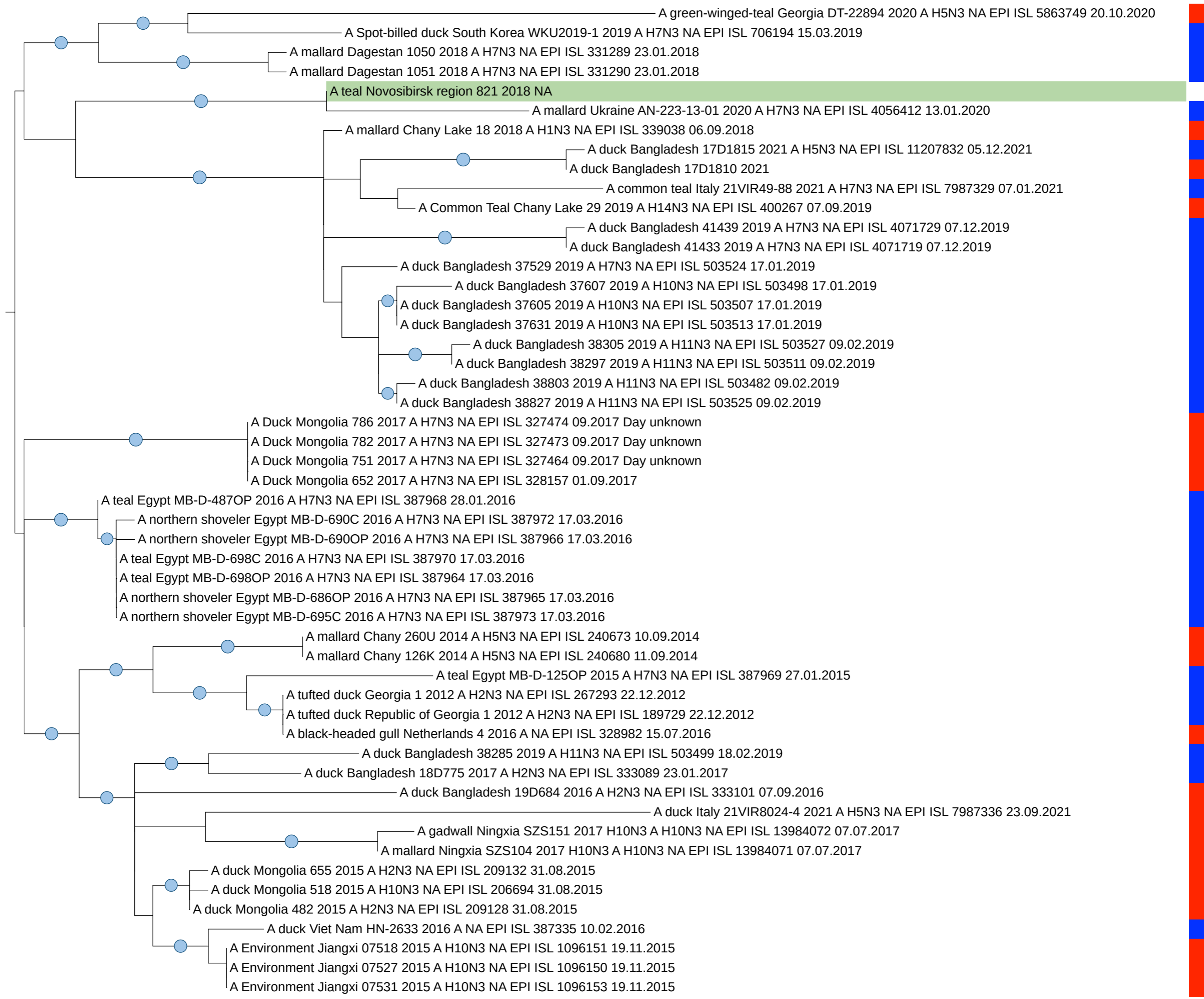

Supplement: Supplementary file 1 [file microorganisms-12-00357-s001.zip › Figure S5.pdf]

Tree scale: 0.01

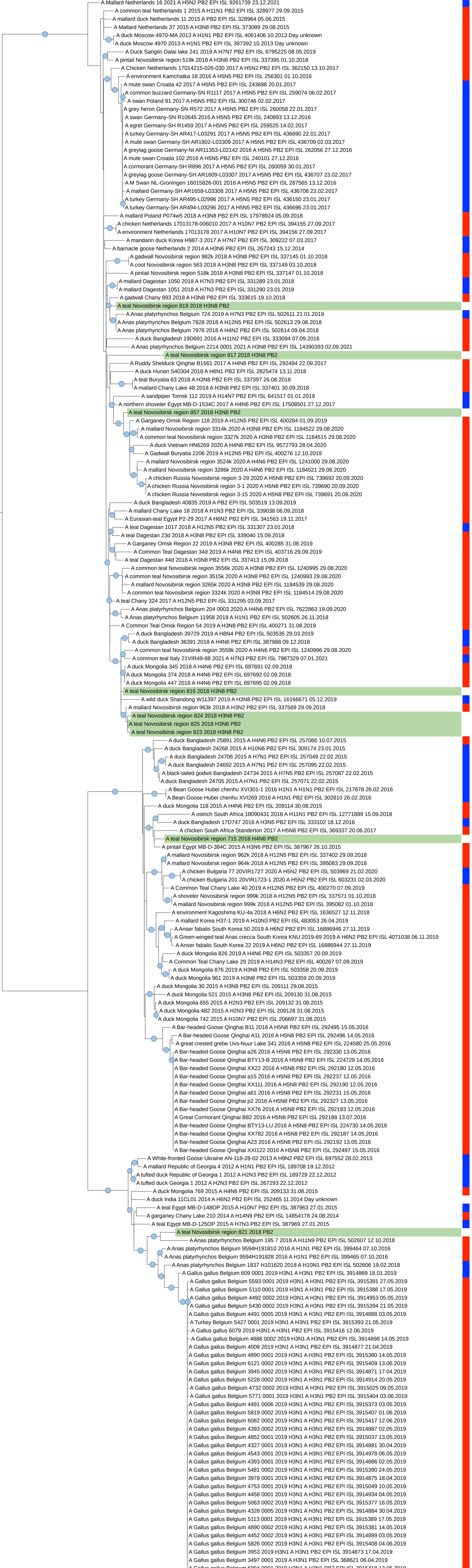

Supplement: Supplementary file 1 [file microorganisms-12-00357-s001.zip › Figure S6.pdf]

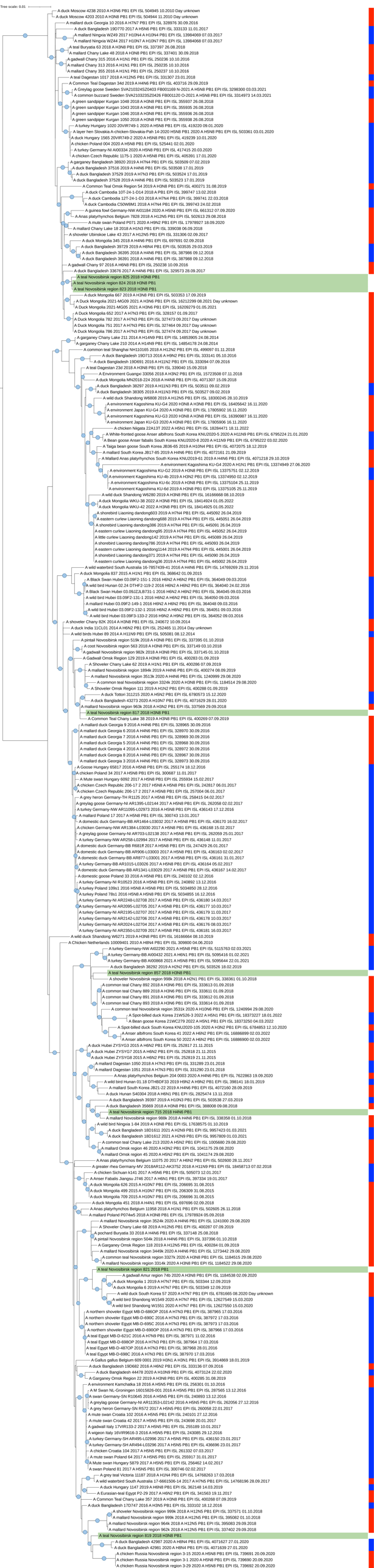

Supplement: Supplementary file 1 [file microorganisms-12-00357-s001.zip › Figure S7.pdf]

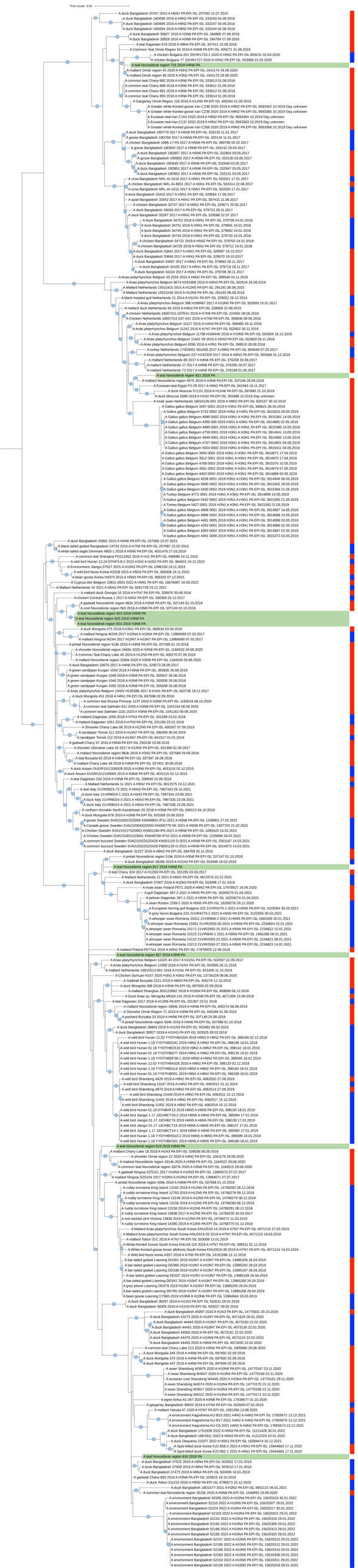

Supplement: Supplementary file 1 [file microorganisms-12-00357-s001.zip › Figure S8.pdf]
